# Hospitalised children with COVID-19 display an aberrant intestinal microbiota and a shift in faecal compounds related with the metabolism of vitamins and lipids

**DOI:** 10.1371/journal.pone.0323910

**Published:** 2025-05-20

**Authors:** Miriam Sanz, Isabel Gutiérrez-Díaz, Hector González, Marta Velasco Rodríguez-Belvís, Rosaura Picáns-Leis, Santiago Jiménez, David González, Jorge Rodríguez, Macarena Queralt, Marta Herrador, Rafael Martín-Masot, Pablo Ferrer, Víctor M. Navas-López, Beatriz Espín, Rosaura Leis, Juan J. Díaz, Susana Delgado

**Affiliations:** 1 Department of Microbiology and Biochemistry of Dairy Products, Instituto de Productos Lácteos de Asturias (IPLA), Consejo Superior de Investigaciones Científicas (CSIC), Villaviciosa, Asturias, Spain; 2 MicroHealth Group, Instituto de Investigación Sanitaria del Principado de Asturias (ISPA), Oviedo, Asturias, Spain; 3 Department of Technology and Biotechnology, Instituto de Productos Lácteos de Asturias (IPLA), Consejo Superior de Investigaciones Científicas (CSIC), Villaviciosa, Asturias, Spain; 4 Gastroenterology and Nutrition Department, Hospital Universitario Infantil Niño Jesús, Madrid, Spain; 5 Hospital Clínico Universitario de Santiago, Santiago de Compostela, Galicia, Spain; 6 Institute of Sanitary Research of Santiago de Compostela (IDIS), CHUS–USC, Santiago de Compostela, Spain; 7 Paediatric Gastroenterology and Nutrition Section, Hospital Universitario Central de Asturias (HUCA), Oviedo, Asturias, Spain; 8 Scientifical and Technic Service, Instituto de Productos Lácteos de Asturias (IPLA), Consejo Superior de Investigaciones Científicas (CSIC), Villaviciosa, Asturias, Spain; 9 Paediatric Gastroenterology Unit, Hospital Universitario Virgen del Rocío de Sevilla, Sevilla, Andalucia, Spain; 10 Paediatric Gastroenterology and Nutrition Unit. Hospital Regional Universitario de Málaga, Málaga, Andalucia, Spain; 11 Paediatric Service, Hospital Universitario y Politécnico La Fe de Valencia, Valencia, Comunidad Valenciana, Spain; Monash University Malaysia, MALAYSIA

## Abstract

The SARS-CoV-2 virus and its rapid spread have made it a global health concern. The aim of this was to investigate the microbial and metabolic faecal profiles of paediatric patients hospitalised for COVID-19 to try to identify biomarkers of predisposition to severity. The study included 16 patients (aged 4–14 years old) from six different Spanish hospitals and 20 age-matched healthy controls. The gut microbiota was characterised by sequencing of 16S rDNA amplicons and internal transcribed space amplicons, while the metabolic profile was analysed by liquid chromatography high resolution mass spectrometry. A different microbial profile was observed between patients and controls, with a significantly higher abundance of sequences belonging to the phyla Bacteroidota and Pseudomonadota in patients. A different metabolic profile was observed between the two groups. Non-infected children had higher faecal levels of vitamins such as niacin, thiamine, and vitamin D3 derivatives, which were negatively correlated with the abundance of pathogenic bacteria, such as members of *Enterobacteriaceae*. Hospitalisation due to SARS-CoV-2 infection in children was associated with changes in the gut microbiota and an altered metabolomic profile. For the first time, several relevant biological compounds were found to be reduced in the faeces of children hospitalised with COVID-19 compared to healthy controls.

## 1. Introduction

The COVID-19 pandemic, caused by the SARS-CoV-2 virus, has affected people of all ages around the world [1]. Although children were initially considered to be a low risk population for severe COVID-19 infection compared to adults, the paediatric population can present with a range of COVID-19 symptoms, from asymptomatic to respiratory and gastrointestinal symptoms [2–4], adding to the complexity of understanding the disease in this population [5,6]. In addition, some children with COVID-19 and gastrointestinal symptoms have been observed to develop more severe complications, although these cases are relatively rare. Approximately 1% of paediatric cases have been found to correspond to more critical diseases such as multisystem inflammatory syndrome (MIS-C), a disease with symptoms similar to Kawasaki disease [5], characterised by gastrointestinal manifestations, systemic inflammation and cardiovascular complications [7,8].

Research into the effects of SARS-CoV-2 in children, particularly those who have required hospitalisation, is essential to improve understanding of the disease and to optimise prevention and treatment strategies. In particular, the gut microbiota, which consists of a variety of beneficial microorganisms that inhabit the gut, plays a critical role in health and the immune system and has been found to be associated with SARS-CoV-2 infection [9,10]. Research linking changes in the microbiota and COVID-19 has been ongoing for several years, although most of the studies have been conducted in adults [11,12], revealing a high presence of opportunistic and pathogenic bacteria [12,13]. In children, some studies suggest that the gut microbiota may influence the immune response to SARS-CoV-2 infection [14–16], and differences in microbiota composition may influence disease severity. However, its precise relationship to disease is not fully understood [15,17].

Metabolomics aims to provide comprehensive metabolite profiling by identifying and quantifying of the complete set of metabolites present in a biological sample. These metabolites include amino acids, lipids, carbohydrates, nucleotides and other compounds smaller than 1,500 Da, and their profile provides valuable information about the physiological state, cellular function, and biochemical interactions in a biological system [18]. Therefore, it may be a promising approach in COVID-19 disease research to study the metabolic changes associated with SARS-CoV-2 infection and its effects on the human body. Studies using metabolomics-based techniques have been conducted to analyse biological samples from patients with COVID-19, including blood, serum, plasma and saliva [19]. These studies have provided valuable information on the metabolic changes associated with the disease, although a full understanding of the relationship between metabolic profiles and COVID-19 disease is still evolving. Applying this strategy to faecal samples and their relationship to the gut microbiota in children with COVID-19 may provide new insights.

The aim of the study was to assess the microbial and metabolomic faecal profiles of a group of paediatric patients hospitalised for COVID-19 and to compare them with a healthy age-matched non-infected group. The identification of potential differential candidate biomarkers associated with the pathology would help to differentiate the role of the disease in terms of its severity and diagnosis.

## 2. Materials and methods

### 2.1. Participants

The study sample included 16 patients infected with SARS-CoV-2, aged 4–14 years, and 20 age-matched healthy controls. Patients were recruited for six public hospitals in Spain: “Hospital Universitario Central de Asturias” (Oviedo),

“Complejo Hospitalario Universitario de Santiago de Compostela” (Santiago de Compostela), “Hospital Universitario y Politécnico La Fe” (Valencia), “Hospital Universitario Infantil Niño Jesús” (Madrid), “Hospital Universitario Virgen del Rocío” (Sevilla), and “Hospital Regional Universitario de Málaga” (Málaga). The recruitment process was carried out over a period of approximately 10 months, from October 2020 to July 2021. The methodology for the collection of faecal samples has been previously published [14]. Inclusion criteria for patient selection were as follows: positive swab test for SARS-CoV-2 using quantitative reverse transcription polymerase chain reaction (RT-qPCR) assay and need for hospital admission. The control group was recruited by paediatricians in Spanish primary care centres at the time of scheduled well-child care visits, and inclusion criteria were as follows; i) no history of antibiotic use during the 3 months prior to sample collection, ii) no clinical history of chronic and/or gastrointestinal diseases, iii) not diagnosed with COVID-19 by RT-qPCR or antigen testing, iv) not in contact with individuals diagnosed or suspected to have SARS-CoV-2 infection, and v) not showing symptoms of COVID-19 at the time of sample collection. Parents or legal guardians of all participants were informed of the aims of the study, and provided written informed consent. Clinical data were collected by clinicians and managed using Research Electronic Data Capture (REDCap) tools [20], hosted at the “Sociedad Española de Gastroenterología, Hepatología y Nutrición Pediátrica” (SEGHNP) (redcap.seghnp.org) with assistance of the AEGREDCap Support Unit, and shared with the “Asociación Española de Gastroenterología” (AEG). This study was approved in accordance with the ethical standards of the Declaration of Helsinki by both the Regional Ethics Committee for Clinical Research (Servicio de Salud del Principado de Asturias, approval number 2000.398) and the Bioethics Committee of the Consejo Superior de Investigaciones Científicas (CSIC) as part of the process of obtaining ethical approval for this research project.

### 2.2. Stool sample preparation

Faecal samples were immediately frozen at -20°C at the recruitment sites after collection. Transport was carried out in accordance with UN3373 standard using CTM03 biological transport containers. Stool samples were thawed on ice for 30 minutes in a biosafety cabinet before processing. Viral inactivation was performed with MagMAX Viral/Pathogen Binding Solution (Thermo Fisher Scientific Inc., Massachusetts, USA) for a period of 10 minutes at room temperature in a 1:2 (w/v) ratio. The treated stool samples were then used for DNA extraction. In addition, 0.5g aliquots of the faecal samples were dissolved in 2ml of methanol, vortexed for 5 minutes, and incubated for 10 minutes at room temperature. The samples were then centrifuged at 13,000 rpm for 15 minutes and the supernatants were stored at -80°C until used for metabolomics analysis.

### 2.3. DNA extraction and quantification

Microbial DNA extraction from faeces was performed according to International Human Microbiome Standards (IHMS) protocol Q, using the QIAamp DNA Stool Mini Kit (Qiagen, Hilden, Germany) with minor modifications [21]. A mechanical lysis was performed in a Fisherbrand Bead Well 24 homogeniser (Thermo Fisher) with three lysis cycles of 45 seconds each, with samples held on ice for five minutes between each treatment. DNA was then eluted and resuspended in 50 µl of molecular biology grade water (Sigma-Aldrich, Saint Louis, USA) and stored at -20ºC until needed. The concentration of the extracted DNA was determined using a Qubit fluorometer with dsDNA assay kits (Thermo Fisher).

### 2.4. High-throughput sequencing of 16S rRNA gene and internal transcribed spacer (ITS) region of bifidobacteria

The 16S rDNA was amplified from the DNA of the samples according to the method described by Milani et al. [22], using the primer pair “Probio_Uni (5′-CCTACGGGRSGCAGCAG-3’)/Probio_Rev” (5′-ATTACCGCGGCTGCT-3’) targeting the V3 region of the 16S rRNA gene. The resulting 250 bp paired-end sequences were obtained using an Illumina MiSeq system (Illumina, San Diego, USA) at the spin-off of the University of Parma Genprobio srl (Italy). Sequence reads were filtered by the Illumina software to remove low-quality sequences. All Illumina quality-assured, trimmed, and filtered sequences were processed using the Quantitative Insights Into Microbial Ecology (QIIME) software suite. After joining the paired-end reads, the quality control phase retained sequences with a mean sequence quality score > 20 and a length between 140 and 400 bp. Sequences with homopolymer regions > 7 bp and those with mismatched primers were omitted. The retained sequences were classified to the lowest possible taxonomic rank (i.e., genus level), with the SILVA database (version 132) serving as the reference. Similarly, the ITS region of the ribosomal RNA genes of the genus *Bifidobacterium* was amplified and subjected to high-throughput sequencing. For this purpose, the primers “Probio_bif_uni” (5′-CTKTTGGGYYCCCKGRYYG-3’) and “Probio_bif_rev” (5′-CGCGTCCACTMTCCAGTTCTC-3’) were used [23], together with an extended bifidobacterial ITS database and Genprobio specific bioinformatic scripts. The raw sequence data were deposited in the Sequence Read Archive (SRA) database of the NCBI (https://www.ncbi.nlm.nih.gov/sra) under BioProject ID PRJNA1083899 (SAMN40261256- SAMN40261291).

### 2.5. Untargeted faecal metabolomics

#### 2.5.1. Sample preparation.

Frozen faecal supernatants were thawed at 4°C and centrifuged at 13,000 rpm for 15 minutes. A volume of 285 µl of sample was mixed with 15 µl of the internal standard solution (500 ppb final concentration of glycoursodeoxycholic acid, C; lauric acid; L-leucine; α-D-glucose; D-lactic acid; L-glutamic acid and cholesterol, all isotopically labelled from Sigma Aldrich) and vortexed for 2 minutes. Samples were then filtered through 0.22 μm PTFE membranes (VWR International, USA) and diluted 1:10 with Milli-Q water. Quality control (QC) samples were prepared by combining pooled samples from each biological replicate, containing an equal volume of all extracts and the internal standard mixture. Samples and QCs were stored at -80°C prior to injection into the liquid chromatography (LC) system,

#### 2.5.2. LC- HRMS instrumental conditions.

A 1290 Infinity II LC system (Agilent Technologies, California USA) coupled to an Agilent 6560B ion mobility quadrupole time-of-flight mass spectrometer (IM-QTOF MS/MS) equipped with an Agilent G1607A dual Jetstream orthogonal electrospray ionisation (ESI) source and the MassHunter WorkStation 11.0, was used operating in positive and negative ion modes in the 50–1000 m/z range. Chromatographic separations were performed on a Zorbax Eclipse plus C18 RR HD column (30x50 mm 1.8 µm, Agilent) at a temperature of 40 ºC and a flow rate of 0.3 ml/min. In positive ion mode, the optimised mobile phases consisted of MilliQ water containing 0.04% formic acid (phase A) and methanol containing 0.04% formic acid (phase B), while in negative ion mode they consisted of water containing 10 mM ammonium formate (Sigma, phase A) and 100% methanol (phase B). The optimised gradient with QCs was set as follows: 5% B for 0.5 min; 5–100% B over 0.5 to 8 min; 100% for 8–12 min; 100–5% B over 12–13 min, re-equilibrating the column for 3 min using the initial solvent composition.

#### 2.5.3. Raw data processing and curation.

All samples were analysed in a randomised order and were interspersed with injections of a QC/filtered blank pair. QCs were injected throughout the run to monitor the performance, stability and reproducibility of the LC-MS method. Data processing was performed using the Profinder software B10.00 (Agilent) according to S1 Table in S1 File. The refined data were exported as CEF files.

#### 2.5.4. Annotation of metabolites and classification.

Preliminary identification of the statistically different metabolites (p ≤ 0.05) was performed using the IDBrowser function of Mass Profiler Professional (MPP, v 15.1) by matching mined features based on accurate mass, isotope ratios, abundances and spacing against metabolites in the METLIN database (http://metlin.scripps.edu), with a mass accuracy window of 5 ppm. Metabolites identities were confirmed by MS/MS analysis, targeting ions by collision-induced dissociation fragmentation. In addition, a database search was carried out to provide a putative classification of the different compounds found in the faecal samples. Several databases such as the Human Metabolome Database (HMDB) [24], the Microbial Metabolites Database (MiMeDB) [25], PubChem [26,27], InterPro [28,29], Chemical Entities of Biological Interest (ChEBI) database [30] and the LIPID MAPS Structure Database (LMSD) [31] were used. Metabolic datasets were converted to mzML format and uploaded to Zenodo (https://zenodo.org/records/15013052 and https://doi.org/10.5281/zenodo.15013051).

### 2.6. Statistical analyses

IBM SPSS Statistics version 28.0.1 (IBM, Armonk, NY, USA) was used as the statistical software for data analysis. Goodness of fit to a normal distribution was analysed using the Kolmogorov-Smirnov test. The U-Mann-Whitney test was used to assess differences between patients and controls in the means of continuous variables (mean ± standard deviation), while categorical variables were examined by the X^2^ test (frequency or percentage). Statistical Principal Coordinates Analysis (PCoA) was performed to assess the similarity between the 16S rDNA sequences of patients and controls. The non-parametric test One-way PERMANOVA was used to determine the statistical significance of the spatial distribution. Supervised and unsupervised statistical analyses of faecal metabolites were performed using MPP software (Agilent, v15.1). Data were s log_2_ converted and scaled, and statistical evaluation of the data was performed using T-test with Benjamini and Hochberg false discovery rate (FDR) correction to adjust the p-values. To further explore the association between differential metabolites and the microbial profile in faecal samples from patients and controls, Spearman correlation method was performed. The relative area of the differential metabolites and the relative abundance of the assigned sequences at phylum and family level were used. The level of statistical significance for all tests was set at p-value ≤ 0.05 and fold-change >1.5.

## 3. Results

### 3.1. Participant characteristics

A total of 16 COVID-19 patients and 20 healthy paediatric controls were included in this study. The mean age of the patients was 10.06 ± 3.17 years, while that of the controls was 10.15 ± 3.13 years. The control group consisted of 45% females and 55% males, while the patients were equally divided between males and females (50%). No significant differences in age and gender were observed between patients and controls.

Focusing on the COVID-19 group, fever was the main reason for hospital admission, although it was not the only symptom reported. Digestive symptoms were reported by 43.75% of the patients with diarrhoea (31.25%), nausea (31.25%), and abdominal pain (25%) being the most common (Table 1). Half of them had respiratory symptoms. Furthermore, there were also three children who developed a more serious complication, the MIS-C (Table 1), two of whom required admission to Intensive Care Units (ICUs).

**Table 1:** Demographic and clinical characteristics of the COVID-19 patients.

Subject code	Age (years)	Clinical history	Days at hospital	Symptoms at admission		
				Fever	Respiratory	Gastrointestinal
Cov-P3	6	–	8	x		
Cov-P6	12	Cystinosis	16	x		
Cov-P7	9	Bronchiolitis obliterans	1	x	x	
Cov-P9	14	–	1	x	x	N + D
Cov-P11^*^	10	–	1	x		AP + N + D
Cov-P14	5	–	7	x		AP + N + D
Cov-P16	13	Juvenile idiopathic arthritis	1			AP + N
Cov-P18	4	Asthma	1		x	
Cov-P20	6	Fanconi’s anemia	15			
Cov-P21	13	Psychiatric disorder	53			
Cov-P23	12	Asthma	180	x		AP + D
Cov-P25^*^	12	–	1	x		D
Cov-P39^*^	11	Bronchiolitis obliterans	1		x	
Cov-P40	10	Metastatic osteosarcoma	1			N
Cov-P42	13	–	1	x		
Cov-P43	11	–	1	x		

-: no relevant information; AP: abdominal pain; N: nausea; D: diarrhoea.

The cases of MIS-C are shown with an asterisk (^*^).

### 3.2. Faecal microbiota compositional profile

**Fig 1:**
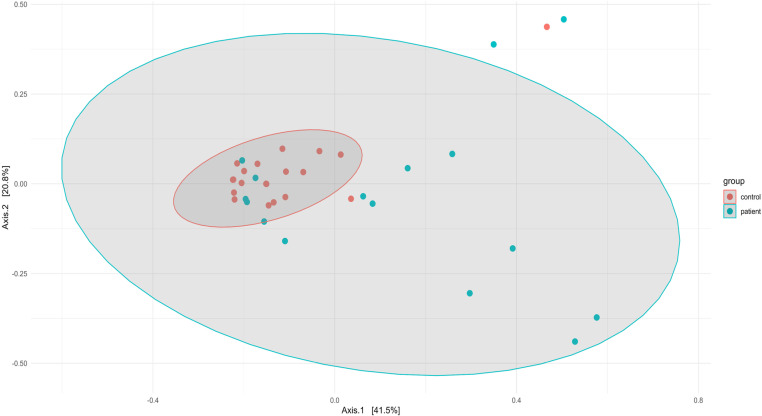
Principal Coordinate Analysis (PCoA). The plot compares the bacterial communities between samples from controls (red circles) and COVID-19 patients (blue circles). The percentages shown on the axes represent the proportion of dissimilarities.

When the microbiota determined by 16S amplicon sequencing was compared between patients and controls, it was observed that both groups clustered separately (Fig 1).

**Fig 2:**
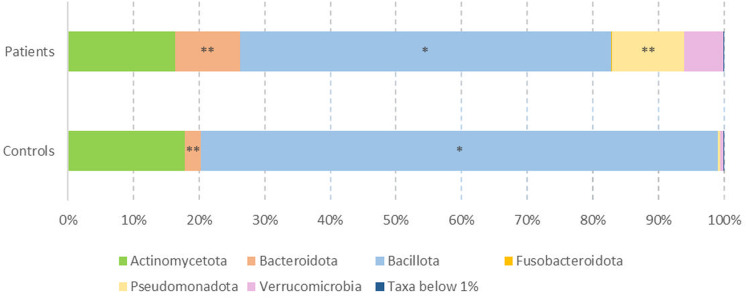
Microbial relative abundances. Differences at the phylum level in faecal samples from COVID patients and controls (* p-value ≤ 0.05; ** p-value ≤ 0.01).

In addition, as it shown in Fig 2, patients had a higher relative abundance of Bacteroidota and Pseudomonadota sequences at phylum level (p-value = 0.004 and p-value < 0.001, respectively). In contrast, Bacillota sequences were reduced compared to controls (p-value = 0.012). Furthermore, the microbial profile in the control group was mainly represented by three phyla (Bacillota, Actinomycetota and to a minor extent Bacteroidota), whereas in the patients, the phyla found were Bacillota and Actinomycetota, followed by Pseudomonadota, Bacteroidota and Verrucomicrobiota.

At the family level, patients had a higher relative abundance of *Enterobacteriaceae* (7.35%), *Bacteroidaceae* (6.31%) and *Tannerellaceae* (2.38%) sequences, whereas the abundances of these families were less than 1% in controls (Table 2). On the other hand, *Prevotellaceae*, *Lachnospiraceae* and *Peptostreptococcaceae* were significantly reduced in patients compared to controls.

**Table 2:** Differences at family level in microbial relative abundance (%) between COVID-19 patients and controls.

Family	Controls (n = 20)	Patients (n = 16)	p-value
*Bifidobacteriaceae*	15.39 ± 14.63	12.79 ± 21.67	0.140
*Coriobacteriaceae*	1.92 ± 1.79	2.44 ± 5.03	0.058
***Bacteroidaceae***	0.95 ± 0.95	6.32 ± 7.15	**0.010**
***Prevotellaceae***	1.09 ± 2.14	0.24 ± 0.75	**0.049**
***Tannerellaceae***	0.19 ± 0.47	2.38 ± 5.58	**0.021**
*Streptococcaceae*	1.13 ± 1.32	3.05 ± 4.86	0.276
*Christensenellaceae*	0.87 ± 0.71	1.11 ± 1.94	0.262
***Lachnospiraceae***	31.98 ± 9.12	18.84 ± 10.48	**<0.001**
***Peptostreptococcaceae***	2.39 ± 2.47	1.15 ± 2.68	**0.003**
*Ruminococcaceae*	37.32 ± 10.04	27.70 ± 18.83	0.109
*Erysipelotrichaceae*	1.00 ± 0.82	0.97 ± 1.20	0.519
*Veillonellaceae*	2.31 ± 2.47	1.25 ± 2.64	0.053
*Idiomarinaceae*	0.01 ± 0.03	3.10 ± 12.40	0.888
***Enterobacteriaceae***	0.19 ± 0.31	7.35 ± 12.90	**<0.001**
*Akkermansiaceae*	0.51 ± 1.17	5.93 ± 11.61	0.789
Others < 1%	2.75 ± 0.26	5.38 ± 0.22	0.067

Mean relative abundance ± standard deviation is represented. Only families with a mean relative abundance above 1% in at least one of the study groups were presented.

**Fig 3:**
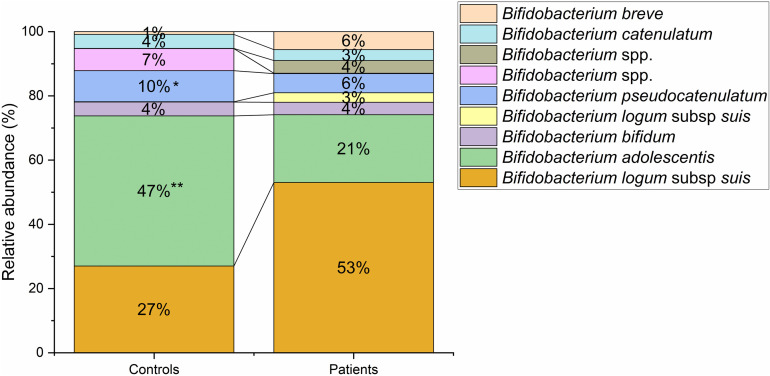
Profiles of bifidobacterial sequences. Data are represented as relative abundance in patients and controls (* p-value ≤ 0.05; ** p-value ≤ 0.01). Data show percentages of *Bifidobacterium* species greater than 1% in at least one group.

Patients had a significantly higher relative abundance of certain genera, including *Bacteroides*, *Parabacteroides*, *Lachnoclostridium* and *Escherichia-Shigella*. Similarly, some genera were reduced in patients compared to controls, such as *Agathobacter*, *Anaerostipes*, *Coprococcus*, *Dorea*, *Fusicatenibacter*, *Lachnospira*, *Tyzzerella*, *Romboutsia*, *Ruminococcus*, *Subdoligranulum* and *Dialister* (S2 Table in S1 File). Notably, seven of these genera with lower relative abundance in patients (p-value ≤ 0.01) belonged to the *Lachnospiraceae* family (*Agathobacter*, *Anaerostipes*, *Coprococcus*, *Dorea*, *Fusicatenibacter*, *Lachnospira*, *Tyzzerella*). Additionally, the ITS amplicons of bifidobacteria were also analysed (Fig 3). The patient group showed a notable decrease in the presence of two species, namely *Bifidobacterium adolescentis* (20.86%) and *B. pseudocatenulatum* (5.90%), when compared to the control group, which had levels of 45.72% and 9.46%, respectively.

### 3.3. Faecal metabolomics profiling

**Fig 4:**
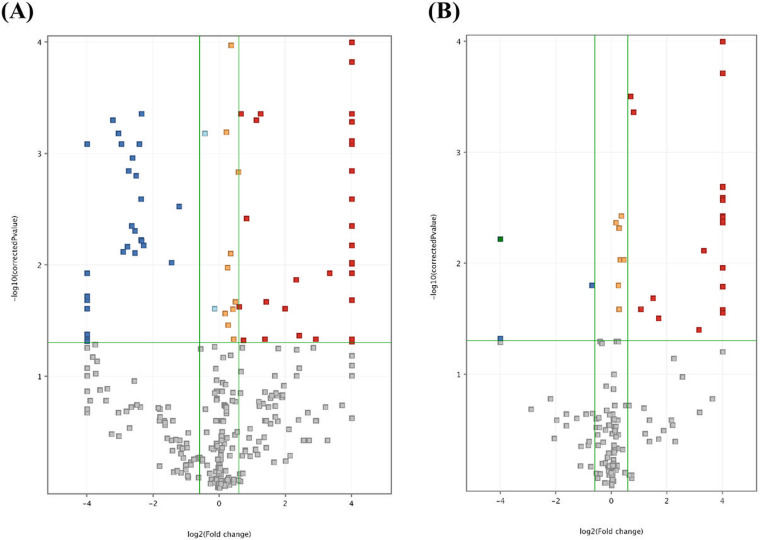
Volcano plot. Differential molecular features comparing control and patient groups in positive (panel A) and negative (panel B) ion modes. Blue and red squares indicate down-regulated and up-regulated compounds, respectively, in controls compared to patients.

Univariate analysis revealed statistically significant differences in the faecal metabolome between patients and controls (Fig 4), with a total of 92 differential molecular features (unpaired t-test). Some of these remained unknown molecules after mass spectrometry (MS/MS) identification, leaving a total of 60 differential metabolites. Of these, 44 were more abundant in controls (S3 Table in S1 File) and 16 in patients (S4 Table in S1 File).

**Fig 5:**
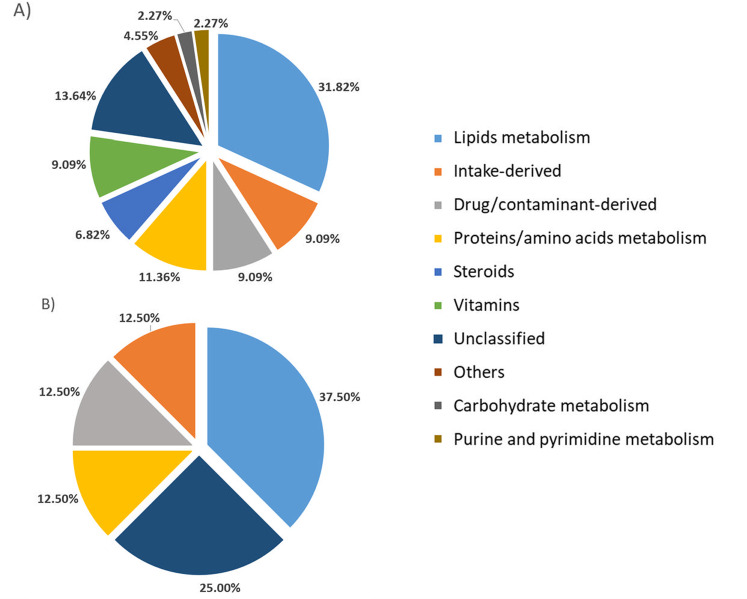
Classification of metabolites. Differential metabolites found in the faeces of controls (A) and patients (B) expressed as percentages.

After a thorough search of the databases, a putative classification was performed, which was able to categorise 83.33% of the 60 metabolites. As shown in Fig 5, the majority of these differential metabolites were related to lipid metabolism (31.82% in controls and 37.5% in patients). In the control group, hydro and liposoluble vitamins (vitamin B_1_, B_3_ and vitamin D_3_ derivatives) were differentially represented. It should be noted that in the control group, 6.82% of the compounds were classified as steroids, which did not appear in the patients.

### 3.4. Metabolites-microbiota associations

**Fig 6:**
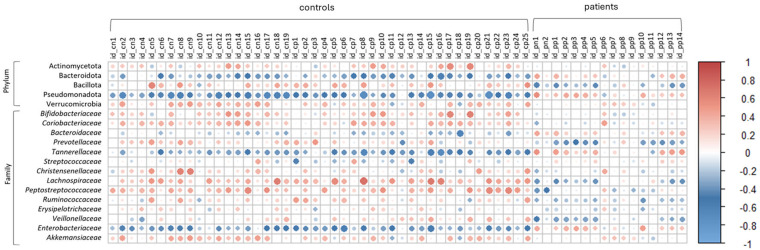
Correlation analysis. Spearman correlation analysis between metabolites and microbiota at phylum and family level. Metabolites codes are shown in Tables S2 and S3 in S1 File.

Spearman correlation was used to determine associations between differentially identified metabolites and the gut microbiota communities at the phylum and family level (Fig 6). It was observed that the strongest correlations (positive or negative) with microbiota were obtained for differentially abundant metabolites present in controls.

Some of these metabolites showed a strong negative correlation with the abundance of Pseudomonadota, particularly with the *Enterobacteriaceae* family of enteric pathogens which had a higher relative abundance in patients. These included thiamine or vitamin B_1_, which was overrepresented in the faeces of controls.

Furthermore, a strong negative correlation was observed between *Tannerellaceae*, a family with pathogenic members of the phylum Bacteroidota, and overrepresented faecal metabolites in the controls, such as 3-indoleacetic acid, valyl-valine and phenylglucuronide. The latter compound was positively associated with the *Lachnospiraceae* family, which was more abundant in the control samples. In addition, a positive correlation was observed between the abundance of *Bifidobacteriaceae* and meta-tyrosine, the steroid epidioxyergosta-diene-diol and lipid metabolism derivatives.

## 4. Discussion

COVID-19 infection is primarily associated with respiratory symptoms, but gastrointestinal symptoms have consistently been shown to be an important component of the disease. A recent meta-analysis of 60 studies found the prevalence of gastrointestinal symptoms in patients with COVID-19 to be 17.6%, with values ranging up to 53%, with discrepancies attributed to regional variations [32]. In children, a German multicentre study found that 17% of hospitalised children reported gastrointestinal symptoms [33]. In Spain, the reported percentages are slightly higher. Gastrointestinal symptoms were observed in 57% of 101 paediatric patients hospitalised with COVID-19 [3]. Our data are consistent with those found in the Spanish population, specifically, 43.75% of our cases had gastrointestinal symptoms.

Several studies have shown that SARS-CoV-2 RNA can persist longer in faecal samples than in nasopharyngeal samples [34,35]. In addition, angiotensin-converting enzyme (ACE2) is expressed not only in the lungs but also in gastrointestinal epithelial cells [36]. These findings may help to explain why some patients experience gastrointestinal symptoms in addition to respiratory symptoms.

Analysis of faecal microbial composition revealed significant differences between SARS-CoV-2 patients and the healthy controls. Hospitalised COVID-19 patients had a microbial profile that was characterised by an abundance of opportunistic organisms, like enterobacteria*,* and a reduction in beneficial commensals, such as *Lachnospiraceae* members. Consistent with previous studies in paediatric patients with COVID-19, our results also indicate a higher abundance of Bacteroidetes (recently renamed Bacteroidota) in the microbiota of patients as compared to controls [15,16,37]. Bacteroidetes have been described to have a mechanism of negative regulation of ACE2 receptors, allowing the virus to enter into cells of the gastrointestinal and respiratory tracts [38,39]. Within this phylum, *Bacteroidaceae* were significantly higher in COVID-19 patients in our study. Islam and colleagues identified *Bacteroides* as a signature genus in the gut of COVID-19 adults with diarrhoea [12]. On the other hand, in our work, patients showed a reduction in the Bacillota (formerly Firmicutes) phylum sequences, which were more abundant in the controls. Previous studies have also reported a decrease in *Lachnospiraceae* and *Ruminococcaceae* in adult patients with COVID-19 [40,41]. Loss of commensal gut microbiota may weaken defences against respiratory pathogens, potentially interacting with SARS-CoV-2 infection. In addition, members of these families that produce short chain fatty acids (SCFAs) may interfere with the host immune response to SARS-CoV-2 infection. In fact, a recent study of vaccinated pregnant women in China revealed a microbiota profile (with members such as *Ruminococcus*), related with SCFAs production, more abundant in women with positive neutralising antibodies and an active immune response [42]. This is not the only study, other research in adults has suggested that faecal microbiota composition and derived metabolites can predict the outcome of patients with severe SARS-CoV-2 infection [43]. We observed a higher abundance of sequences belonging to the phylum Pseudomonadota (formerly Proteobacteria) in our COVID-19 patients. A higher presence of Proteobacteria in SARS-CoV-2 patients has been demonstrated in several studies [12,44], including paediatric populations [14,45]. Indeed, an increase in microorganisms belonging to the *Enterobacteriaceae* family has been observed in respiratory influenza infections [46].

The faecal metabolomic profile showed significant differences between the two groups. Metabolites associated with key functional pathways were found to be upregulated in the control group. Two B vitamins, vitamin B_1_ (thiamine) and vitamin B_3_ (niacin), both associated with proper immune function, were over-represented in the faeces of the control group. Niacin reduces neutrophil infiltration, has anti-inflammatory effects, and reduces viral replication [47]. Recent research has shown that vitamin B_1_ deficiency is associated with a variety of biological changes at the metabolic, neurological, cardiovascular, respiratory, gastrointestinal and skeletal muscle levels [48]. Therefore, maintaining adequate levels of thiamine may support a proper immune response during SARS-CoV-2 infection. Although the primary source of vitamin B_1_ is dietary, many bacteria in the gut are able to synthesise vitamin B_1_, while others are not [49]. In particular, adequate dietary vitamin B_1_ intake affects the relative abundance of *Ruminococcaceae*, a family belonging to the phylum Bacillota. These bacteria require dietary vitamin B_1_ because they lack the synthetic de novo vitamin B_1_ pathway [50]. Our results indicate a negative correlation between faecal vitamin B_1_ and the abundance of enterobacteria.

The infants in the control group had a statistically higher frequency of products resulting from vitamin D_3_ metabolism in their faeces compared to the patients. Several studies have reported an inverse correlation between vitamin D_3_ levels and COVID-19 infection [51,52]. Vitamin D_3_ is also known to have anti-inflammatory properties, particularly in viral infections. It is also involved in the production of glutathione reductase, a powerful antioxidant [51]. In this regard, another metabolite related to the glutathione cycle, (R)-(+)-2-pyrrolidone-5-carboxylic acid, was more abundant in the control group of our study. Glutathione is an important antioxidant molecule that fights reactive oxygen species. Some studies have suggested that certain viral infections may lead to depletion of this molecule [53]. In addition, SARS-CoV-2 infection has been shown to inhibit the transcription of certain factors, including the vitamin D_3_ receptor [53]. Indeed, vitamin D and its receptor (VDR) signalling have been proposed to contribute to genetic, environmental, immune, and microbial aspects of several human diseases [54]. In addition, another metabolite that was found to be over-represented in the faeces of the controls was 3-indoleacetic acid. This is an indole derivative that may promote intestinal barrier integrity and suppress inflammatory responses [55]. Lower serum levels of this metabolite were observed in hospitalised adults with SARS-CoV-2 infection compared to healthy controls [56].

Although some studies have suggested that diet, including vitamins, may assist the immune system in combating SARS-CoV-2 infection, it is essential to consider the complex relationship between the microbiota and its derived metabolites in order to improve our understanding of COVID-19 pathophysiology. Lipid metabolism and endogenous steroids are important for the normal physiology, full development and growth of children during these ages. Any nutritional deficiencies in vitamins, amino acids, or hormonal alterations would increase the risk of infection and further complications, including hospitalisation. Several studies have shown that lower vitamin D levels are associated with a high risk of acute respiratory infections [57,58]. However, the acute phase of infection and hospitalisation can considerably influence metabolic profiles and vitamin levels, regardless of pre-existing nutritional status. Acute illness can lead to transient changes in nutrient levels due to factors such as inflammation, altered intake, and metabolic demands [59]. As our study is exploratory and observational, we cannot distinguish whether the results and associations found are a consequence or a contributing factor to SARS-CoV-2 infection and hospitalisation. As a major limitation, the study was underpowered due to the small sample size which limits the generalisability of our findings. As a strength, there is very little research on metagenomics and metabolomics in scholars and preadolescents with COVID-19. Our findings provide an important preliminary basis for understanding paediatric SARS-CoV-2 infection. Future studies with large cohorts are needed to confirm our findings and improve generalisation.

## Conclusions

This study is the first to assess the gut microbial profile and use untargeted faecal metabolomics to identify differential intestinal metabolites between hospitalised children with COVID-19 and a control group. Our results suggest that COVID-19 has been associated with gastrointestinal symptoms and altered gut microbiota. Intriguingly, vitamins B_1_ and D_3_ derivatives, important in combating infections shower less abundance in infected children. Proper nutrition and a healthy gut microbiota are crucial for a strong immune response, particularly in children. It is our hope that this work will facilitate the identification of novel research targets for the future, linking diet, functional gut microbiota and response to global health concerns such as those caused by SARS-CoV-2 during the pandemic.

## Supporting information

S1 File S1 Table. Batch Recursive Feature Extraction parameters applied for metabolomics data processing and curation. **S2 Table.** Statistically significant differences at genus level in microbial relative abundance (%) between COVID-19 patients and controls shown as mean relative abundance ± standard deviation. **S3 Table.** Classification of the 44 differential major metabolites found in faeces of controls. **S4 Table.** Classification of the 16 differential major metabolites found in faeces of patients in positive (second p in the letter code) and negative (n in the letter code) ion mode.
(DOCX)
