# Supplementary material for: Hospitalised children with COVID-19 display an aberrant intestinal microbiota and a shift in faecal compounds related with the metabolism of vitamins and lipids
Source: PLoS One. 2025 May 20;20(5):e0323910. doi: 10.1371/journal.pone.0323910 (PMC12091793; doi:10.1371/journal.pone.0323910)
Supplement: S1 File — Batch Recursive Feature Extraction parameters applied for metabolomics data processing and curation. S2 Table. Statistically significant differences at genus level in microbial relative abundance (%) between COVID-19 patients and controls shown as mean relative abundance ± standard deviation. S3 Table. Classification of the 44 differential major metabolites found in faeces of controls. S4 Table. Classification of the 16 differential major metabolites found in faeces of patients in positive (second p in the letter code) and negative (n in the letter code) ion mode. (DOCX) [file pone.0323910.s001.DOCX]

## SUPLEMENTARY INFORMATION

**S1 Table.** Batch Recursive Feature Extraction parameters applied for metabolomics data processing and curation.

| **Batch Recursive Feature Extraction** | |
| --- | --- |
| **Extraction** | |
| Peak weight | > 1200 counts (Positive mode)  >3500 counts (Negative mode) |
| **Ion species** | |
| Positive Mode | [M+H]^+^, [M+Na]^+^ |
| Negative Mode | [M-H]^-^ , [M-HCOO]^-^ |
| **Charge State** | 1-2 |
| **Mass filters (excluded)** | 121.0509 (ESI^+^); 922.0098(ESI^+^); 119.0363 (ESI^-^); 963.0007 (ESI^-^) |
| **Integration** | Agile 2 |
| **Alignment Parameters** | |
| RT Tolerance | 0.00% ± 0.15 min |
| Mass Tolerance | 5 ppm ± 2.00 mDa |
| **Molecular Feature Extraction Filters** | |
| Score (MFE) | > 80 |
| **Tolerance and EIC** | |
| Masses | ± 5.00 ppm |
| RT | ± 0.150 min |
| Possible m/z | Symmetric (ppm) |
| **Peak Filter** | |
| Absolute height | > 20000 counts |
| **Chromatogram Format** | Centroid |
| **Peak Spectrum** | |
| Average scans | 10 % of peak height |
| Exclude if above | 20% of saturation |
| **Find by ion filters** | |
| Score (Tgt) | > 80 |
| **Entities** | |
| Number of entities found | Positive Mode: 3097 |
|  | Negative Mode: 1631 |

**S2 Table.** Statistically significant differences at genus level in microbial relative abundance (%) between COVID-19 patients and controls shown as mean relative abundance ± standard deviation.

| **Genera** | **Controls (n=20)** | **Patients (n=16)** | **p value** |
| --- | --- | --- | --- |
| *Bacteroides* | 0.95 ± 0.94 | 6.32 ± 7.14 | 0.010 |
| *Parabacteroides* | 0.19 ± 0.47 | 2.38 ± 5.58 | 0.021 |
| *Agathobacter* | 4.03 ± 3.66 | 0.25 ± 0.52 | 0.001 |
| *Anaerostipes* | 1.31 ± 0.90 | 0.54 ± 1.14 | 0.002 |
| *Coprococcus* sp. | 1.42 ± 0.95 | 0.61 ± 1.01 | 0.005 |
| *Dorea* | 1.12 ± 0.73 | 0.26 + 0.52 | 0.001 |
| *Fusicatenibacter* | 1.68 ± 1.33 | 0.35 ± 0.65 | 0.001 |
| *Lachnospira* | 0.65 ± 1.13 | 0.23 ± 0.73 | 0.004 |
| *Tyzzerella* | 1.14 ± 0.76 | 0.63 ± 1.26 | 0.007 |
| *Romboutsia* | 2.36 ± 2.38 | 0.98 ± 2.19 | 0.002 |
| *Ruminococcus* | 2.04 ± 2.01 | 0.81 ± 1.45 | 0.018 |
| *Subdoligranulum* | 4.59 ± 4.67 | 1.29 ± 1.94 | 0.001 |
| *Dialister* | 1.78 ± 2.35 | 0.71 ± 2.22 | 0.033 |
| *Escherichia_Shigella* | 0.12 ± 0.26 | 5.70 ± 12.21 | 0.021 |

Only genera with a mean relative abundance above 1% in at least one of the study groups are represented.

**S3 Table**. Classification of the 44 differential major metabolites found in faeces of controls.

| **METABOLITES (N)** | **CODE** | **CLASSIFICATION** |
| --- | --- | --- |
| 7beta,12beta-Dihydroxy-5alpha-cholan-24-oic Acid | id-cn1 | Lipids metabolism |
| 11beta-Hydroxy-5beta-cholan-24-oic Acid | id-cn2 | Lipids metabolism |
| Chatenaytrienin 2 | id-cn3 | Lipids metabolism |
| Hexadecanoic acid, 8-hydroxy-, (S)-; Hexadecanoic acid, 8-hydroxy-, (+)- | id-cn4 | Lipids metabolism |
| L-Urobilin | id-cn5 | Others (porphyrins) |
| 6-Imino-5-oxocyclohexa-1,3-dienecarboxylate | id-cn6 | Unclasiffied |
| PG(P-20:0/17:1(9Z)) | id-cn7 | Lipids metabolism |
| Enterolactone | id-cn8 | Intake-derived |
| Furfuryl isovalerate | id-cn9 | Lipids metabolism |
| CerP(d18:1/20:0) | id-cn10 | Lipids metabolism |
| 1,1'-Thiobisethanethiol | id-cn11 | Unclasiffied |
| Ximaosteroid C | id-cn12 | Steroid |
| 1α,25-dihydroxy-2β-(2-hydroxyethoxy)vitamin D3 / 1α,25-dihydroxy-2β-(2-hydroxyethoxy)cholecalciferol | id-cn13 | Vitamin |
| 1α,25-dihydroxy-21-nor-20-oxavitamin D3 / 1α,25-dihydroxy-21-nor-20-oxacholecalciferol | id-cn14 | Vitamin |
| Methyl 2,3-dihydro-3,5-dihydroxy-2-oxo-3-indoleacetic acid | id-cn15 | Others (Indoles) |
| [6]-Gingerdiol 3,5-diacetate | id-cn16 | Lipids metabolism |
| Ethylmethylmaleimide | id-cn17 | Lipids metabolism |
| Norselic acid E | id-cn18 | Steroid |
| (R)-(+)-2-Pyrrolidone-5-carboxylic acid | id-cn19 | Protein metabolism |

N: detected in negative ion mode

**continues S3 Table …**

| **METABOLITES (P)** | **CODE** | **CLASSIFICATION** |
| --- | --- | --- |
| Methacholine | id-cp1 | Protein metabolism |
| N-oleoyl phenylalanine | id-cp2 | Lipids metabolism |
| 5-alpha-cholesta-7,24-dien-3-beta-ol | id-cp3 | Lipids metabolism |
| Adlupulone | id-cp4 | Drug/contaminant-derived |
| Nicotinic Acid | id-cp5 | Vitamin |
| N-stearoyl tyrosine | id-cp6 | Lipids metabolism |
| 3-Carboxypropyl trimethylammonium | id-cp7 | Unclasiffied |
| Phenyl glucuronide | id-cp8 | Carbs. metabolism |
| Methyl 3b,24-dihydroxy-11,13(18)-oleanadien-30-oate | id-cp9 | Lipids metabolism |
| Schleicherastatin 3 | id-cp10 | Lipids metabolism |
| Xanthine | id-cp11 | Purine and pyrimidine metabolism |
| Pyrrolidine | id-cp12 | Unclasiffied |
| Butethal | id-cp13 | Drug/contaminant-derived |
| Thiamine | id-cp14 | Vitamin |
| 1,6-Anhydro-N-acetyl-beta-muramate | id-cp15 | Intake-derived |
| Valyl-Valine | id-cp16 | Intake-derived |
| (3beta,5alpha,6beta,9alpha,22E,24R)-5,9-Epidioxyergosta-7,22-diene-3,6-diol | id-cp17 | Steroid |
| (R)-(+)-2-Pyrrolidone-5-carboxylic acid | id-cp18 | Protein metabolism |
| Meta-Tyrosine | id-cp19 | Protein metabolism |
| Methylmethionine sulfonium salt | id-cp20 | Unclasiffied |
| Zeaxanthin | id-cp21 | Intake-derived |
| N-Hydroxy-L-phenylalanine | id-cp22 | Protein metabolism |
| Isocarbamid | id-cp23 | Drug/contaminant-derived |
| 3-Hydroxy-N-methylpyridinium | id-cp24 | Drug/contaminant-derived |
| N4-Phosphoagmatine | Id-cp25 | Unclasiffied |

P: detected in positive ion mode

**S4 Table**. Classification of the 16 differential major metabolites found in faeces of patients in positive (second p in the letter code) and negative (n in the letter code) ion mode.

| **METABOLITES** | **CODE** | **CLASSIFICATION** |
| --- | --- | --- |
| 26-hydroxycholesterol 3-sulfate | id-pn1 | Lipids metabolism |
| Lauryl hydrogen sulfate | id-pn2 | Unclasiffied |
| Arg Gln Arg | id-pp1 | Proteins metabolism |
| PA(20:5(5Z,8Z,11Z,14Z,17Z)/22:6(4Z,7Z,10Z,13Z,16Z,19Z)) | id-pp2 | Lipids metabolism |
| Hebevinoside XIII | id-pp3 | Unclasiffied |
| PE(22:6(4Z,7Z,10Z,13Z,16Z,19Z)/18:4(6Z,9Z,12Z,15Z)) | id-pp4 | Lipids metabolism |
| Morellinol | id-pp5 | Lipids metabolism |
| 1,4-Methylimidazoleacetic acid | id-pp6 | Intake-derived |
| Hexadecanoic acid, 2-amino-, (1)- | id-pp7 | Lipids metabolism |
| N,N,N-Trimethylmethanaminium | id-pp8 | Unclasiffied |
| 10-F2-dihomo-IsoP | id-pp9 | Lipids metabolism |
| 3',4'-(Methylenedioxy)acetophenone | id-pp10 | Intake-derived |
| 2-Benzofurancarboxaldehyde | id-pp11 | Unclasiffied |
| PD-160725 2-hydroxyethanesulfonate | id-pp12 | Drug/contaminant-derived |
| Arg Ser Ser | id-pp13 | Proteins metabolism |
| HC Toxin | id-pp14 | Drug/contaminant-derived |
